# Peripheral blood mononuclear cells contribute to myogenesis in a 3D bioengineered system of bone marrow mesenchymal stem cells and myoblasts

**DOI:** 10.3389/fbioe.2022.1075715

**Published:** 2023-01-10

**Authors:** Pasqualina Scala, Paola Manzo, Erwin Pavel Lamparelli, Joseph Lovecchio, Maria Camilla Ciardulli, Valentina Giudice, Carmine Selleri, Emanuele Giordano, Laura Rehak, Nicola Maffulli, Giovanna Della Porta

**Affiliations:** ^1^ Department of Medicine, Surgery and Dentistry, University of Salerno, Baronissi, Italy; ^2^ Hematology and Transplant Center, University Hospital “San Giovanni di Dio e Ruggi D’Aragona”, Salerno, Italy; ^3^ Department of Electrical, Electronic and Information Engineering “Guglielmo Marconi” (DEI), University of Bologna, Bologna, Italy; ^4^ Athena Biomedical innovations, Florence, Italy; ^5^ Centre for Sports and Exercise Medicine, Barts and The London School of Medicine and Dentistry, Queen Mary University of London, London, England; ^6^ Interdepartment Centre BIONAM, University of Salerno, Fisciano, Italy

**Keywords:** myogenesis, tissue engineering, mesenchymal stem cells, peripheral blood mononuclear cells, 3D Co-culture model

## Abstract

In this work, a 3D environment obtained using fibrin scaffold and two cell populations, such as bone marrow-derived mesenchymal stem cells (BM-MSCs), and primary skeletal muscle cells (SkMs), was assembled. Peripheral blood mononuclear cells (PBMCs) fraction obtained after blood filtration with HemaTrate^®^ filter was then added to the 3D culture system to explore their influence on myogenesis. The best cell ratio into a 3D fibrin hydrogel was 1:1 (BM-MSCs plus SkMs:PBMCs) when cultured in a perfusion bioreactor; indeed, excellent viability and myogenic event induction were observed. Myogenic genes were significantly overexpressed when cultured with PBMCs, such as *MyoD1* of 118-fold at day 14 and *Desmin* 6-fold at day 21. Desmin and Myosin Heavy Chain were also detected at protein level by immunostaining along the culture. Moreover, the presence of PBMCs in 3D culture induced a significant downregulation of pro-inflammatory cytokine gene expression, such as *IL6*. This smart biomimetic environment can be an excellent tool for investigation of cellular crosstalk and PBMC influence on myogenic processes.

## 1 Introduction

*In vitro* skeletal muscle regeneration (SkMR) models are extremely challenging to reproduce because resident satellite cells (SCs) - that mediate the entire process *in vivo*-are very difficult to harvest from muscle tissue, and lose their engraftment potential in *ex vivo* conditions with progressive growth rate reduction (Montarras et al., 2005; Kuang et al., 2007; Charville et al., 2015; Garcia et al., 2017). Bone marrow-derived mesenchymal stem cells (BM-MSCs) represent the gold standard for musculoskeletal regeneration study and research (Ciardulli et al., 2020; Ciardulli et al., 2021; Lamparelli et al., 2021). They were also proposed as a promising alternative in SkMR because of their ability to differentiate towards various lineages, low immunogenicity, and high immunomodulatory activity (Vater et al., 2011; Shi et al., 2018). However, BM-MSCs are still poorly investigated for *in vitro* myogenesis purpose because of the lack of standardized medium composition and appropriate culture system approaches, although BM-MSCs co-cultured with myoblasts show significant upregulation of muscle markers, such as *Myogenic Regulatory Factors* (*MRFs*), *Desmin*, and *Myosin heavy chain II* (*MYH2*) (Beier et al., 2011; Scala et al., 2022).

Myogenesis is also strongly influenced by cytokines secreted by peripheral blood mononuclear cells (PBMCs) involved in tissue regenerative processes, like clearance of necrotic debris, inflammation, and remodeling processes (Scala et al., 2021). Moreover, pro-inflammatory cytokines, including interferon-γ (IFN-γ), tumor necrosis factor (TNF), interleukin (IL)-6 and IL-1, released by PBMCs influence SkMR fate by driving the first phase of repair process and by modulating immune responses and SC activation (Chen et al., 2007; Cheng et al., 2008; Londhe and Davie, 2011; Belizário et al., 2016; Chaweewannakorn et al., 2018). Conversely, anti-inflammatory cytokines, especially IL-10, are associated with the transition from proliferative to differentiation phase of myogenesis (Villalta et al., 2011; Deng et al., 2012). Indirect evidence of the essential role of PBMCs in SkMR is the efficacy of autologous transplantation of peripheral blood cells isolated by commercial blood filtration systems for treatment of critic limb ischemia by increasing regenerated myofibers and by improving clinical outcomes (Spaltro et al., 2015; Rigato et al., 2017).

On the other hand, biomimetic 3D cultures of stem cell represent a versatile tool for the study of myogenic commitment events (Witt et al., 2017; Ergene et al., 2020). Indeed, BM-MSCs are frequently cultured in 3D hydrogel scaffolds, like fibrin hydrogels (Govoni et al., 2017; Ciardulli et al., 2020; 2021; Manzo et al., 2022) that efficiently mimic SC niche (Pollot et al., 2018) and may reproduce *in vitro* the hierarchical structure of skeletal muscle tissue. Moreover, fibrin-based scaffolds constitute a regenerative myogenic environment without fibrosis, as cells produce their own extracellular matrix (ECM) proteins and degraded fibrin excess allowing long-term skeletal muscle cell culture (Huang et al., 2005; Gilbert-Honick et al., 2018; Matthias et al., 2018). When a perfusion bioreactor system is adopted for these 3D *in vitro* cultures, the medium easily diffuses through the 3D structure enhancing metabolite mass transfer, nutrient transport, and oxygenation rate. In addition, 3D perfused cultures show excellent viability, high proliferation rates, and more efficient commitment processes (Grayson et al., 2011; Birru et al., 2018; Pasini et al., 2019; Engel et al., 2021; Lamparelli et al., 2021).

In this study, we aimed to develop an *in vitro* model of myogenic commitment using human BM-MSCs (*h*BM-MSCs) and human primary skeletal myoblasts (*h*SkMs) cultured in 3D dynamic conditions assembled within a fibrin scaffold. Moreover, PBMCs were added within the same 3D co-culture to investigate their activity on *h*BM-MSC myogenic commitment. To our knowledge, 3D *in-vitro* models studying the effects of PBMCs on stem cell commitment has been proposed only in osteogenic events (Gamblin et al., 2014; Tang et al., 2019); whereas, 3D dynamic co-cultures of BM-MSCs and PBMCs applied to SkMR are not reported yet. *h*BM-MSC myogenic events evolution was performed by qRT-PCR and immunohistochemistry, whereas, PMBCs characterization was performed by flow cytometry.

## 2 Materials and methods

### 2.1 PBMC concentration and harvesting

PBMCs were collected by HemaTrate^®^ Blood Filtration System, following same protocol of clinical practice in limb rescue (De Angelis et al., 2015; Persiani et al., 2018), Whole peripheral blood (PB) obtained from three healthy volunteers (M/F, 2/1; age ranged from 38 to 45 years old) was concentrated using a HemaTrate^®^ Blood Filtration System (CH-WB110C, Patent n. EP 2602315A1; Pall Medistad B.V. (Medemblik; Netherlands) from Cook Regentec (Indianapolis, Indiana, United States). After filtration, PBMCs were isolated by Ficoll-Paque density gradient centrifugation (Cytiva, Marlborough, Massachusetts, United States), according to manufacturer’s instructions, for flow cytometer immunophenotyping. Conversely, filtrated PBMCs were directly used for 3D culture experiments, or were stored in 70% RPMI (Gibco™), 20% Fetal Bovine Serum (FBS, Gibco™), and 10% DMSO (Sigma-Aldrich, Milan, Italy) at -80°C until use.

### 2.2 *h*BM-MSC isolation, harvesting, and characterization

BM specimens for *h*BM-MSC isolation were obtained from three healthy male donors (aged 26, 24, and 28 years old) after informed written consent in accordance with the Declaration of Helsinki and protocols approved by our Institutional Review Board (Ethic Committee “Campania Sud”, Brusciano, Naples, Italy; prot./SCCE n. 24,988). BM aspirate culture was described elsewhere (Giordano et al., 2014). BM-MSCs were subsequently seeded at 4,000 cells/cm^2^ and expanded up to the third passage. Mesenchymal phenotype was confirmed according to the International Society of Cellular Therapy guidelines: i) ability to adhere to tissue culture plastics; ii) fibroblast-like spindle shape; and iii) characteristic immunophenotype by flow cytometry with positivity for CD90, CD105, and CD73, and negativity for CD34, CD14, CD45, and HLA-DR (Supplementary Figure S1) (Dominici et al., 2006). *h*BM-MSCs were co-cultured in a 3D system with *h*SkMs, with and without PBMCs. All experiments were performed in biological triplicates (N = 3).

### 2.3 *h*SkM characterization

*h*SkMs were purchased from (Gibco™). *h*SkMs were embedded in fibrin scaffolds to investigate their behavior in a 3D environment through qRT-PCR, as reported in the following section. Then, *h*SkMs were co-cultured with *h*BM-MSCs with and without PBMCs. Seeding ratio between *h*BM-MSCs:*h*SkMs was 2:1, as previously optimized in (Scala et al., 2022). All experiments were performed in biological triplicates (N = 3).

### 2.4 Flow cytometry

MSC and PBMC immunophenotype was investigated by flow cytometry. Briefly, for BM-MSCs, a minimum of 1 × 10^5^ cells at the third passage was stained with the following antibodies: 2.5 μl of fluorescein isothiocyanate (FITC) - conjugated anti-CD90 or 5 μl of FITC - conjugated anti-HLA-DR; 5 μl of allophycocyanin (APC) - conjugated anti-CD73; 10 μl of phycoerythrin (PE) - conjugated anti-CD105 or 10 μl of PE - conjugated anti-CD34; and 10 μl of phycoerythrin cyanin 7 (PC7) - conjugated anti-CD45 or 10 μl of PC7 - conjugated anti-CD14 (all antibodies from Beckman Coulter, Fullerton, CA, United States). Cells were incubated at room temperature (RT) for 20 min in the dark, washed with phosphate buffered saline (PBS, Gibco™), and resuspended in 300 μl of the same buffer for acquisition. For PBMC immunophenotyping, before and after filter and separation procedures, a minimum of 2 × 10^5^ cells were stained with the following antibodies: 5 μl of APC–conjugated anti-CD3; 5 μl PC7 - conjugated anti-CD14; 5 μl of FITC–conjugated CD34; 5 μl of phycoerythrin cyanin 5 (PC5)—conjugated CD8; and 5 μl of PE - conjugated anti-CD4. Cells were then incubated at RT for 20 min in the dark, washed with PBS and resuspended in 300 μl of the same buffer for acquisition. Sample acquisition was performed on a BD FACSVerse flow cytometer (Becton Dickinson, BD, NJ, United States) equipped with blue (488 nm) and red lasers (628 nm) and BD FACSuite software (BD Biosciences). PMT voltage setting, and compensation were carried out using single-color controls for each fluorochrome and an unstained sample as negative control. All samples were run with the same PMT voltages, and a minimum of 30,000 events were recorded. FlowJo software (v.10.7.1, LLC, BD Biosciences) was employed for post-acquisition compensation and analysis.

BM-MSCs were first identified using linear parameters (forward scatter area (FSC-A) vs*.* side scatter area (SSC-A), and double cells were excluded (area vs*.* height, FSC-A vs*.* FSC-H) (Supplementary Figure S1).

### 2.5 Scaffold drying and FE-SEM analysis

**FIGURE 1:**
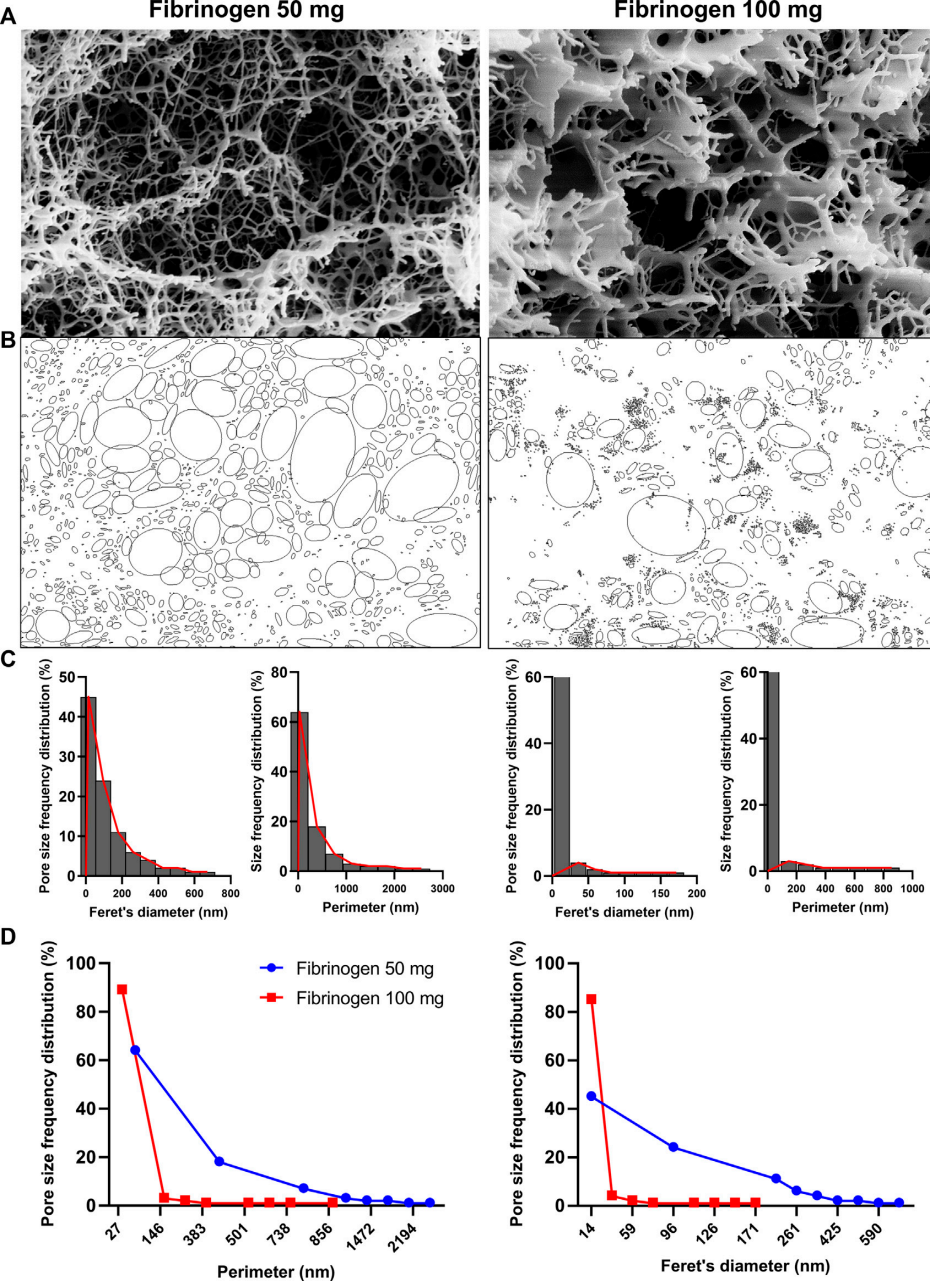
Field Emission-Scanning electron microscopy (FE-SEM) images of aerogels and of their internal structure morphology at two tested fibrinogen concentrations, 100 mg/ml and 50 mg/ml scaffolds were dried by dense gas in order to avoid 3D structure shrinkage of aerogel that was clearly observed after scaffold freeze-fracturing in liquid nitrogen **(A);** Perimeter and Feret’s diameter values were calculated with ImageJ software, and pores were automatically determined after threshold adjustment in 100 mg/ml and 50 mg/ml fibrinogen scaffolds **(B)**. Size frequency distributions are shown for perimeter and Feret’s diameter values in 100 mg/ml and 50 mg/ml fibrinogen scaffolds **(C),** and **(D)** compared between the two concentrations.

Cylindrical 3D scaffolds were prepared by mixing fibrinogen at 50 or 100 mg/ml (Sigma-Aldrich), α-aprotinin 15,600 U/ml (Sigma-Aldrich), α-MEM (Corning, NY, United States), and thrombin at 100 U/ml (Sigma-Aldrich), and then were incubated at 37°C for 30 min to allow fibrinogen polymerization (Supplementary Figure S2A). Samples were fixed in 4% paraformaldehyde (PFA; 4°C, overnight) and then dehydrated by multiple passages across ethanol: water solutions (10 min each) with increasing percentages of ethanol and dried using a dense carbon dioxide drying operating at 200 bar and 38°C for 4 h; other details on the dense gas drying protocol were reported elsewhere (Della Porta et al., 2013). System technology scheme is illustrated in Supplementary Figure S2B. Samples were immersed in liquid nitrogen and fractured with a needle, then placed on a double-sided adhesive carbon tape previously glued to an aluminium stub and coated with a gold film (250 A thickness) using a sputter coater (mod.108 A; Agar Scientific, Stansted, United Kingdom), before observation. Internal scaffold morphology was observed by field emission-scanning electron microscopy (FE-SEM, mod. LEO 1525; Carl Zeiss, Oberkochen, DE). Pore size frequency distribution was studied with ImageJ software (rel.1.52p National Institutes of Health, United States), and average perimeter and average Feret’s diameter values were reported (Figure 1).

### 2.6 3D bioengineered scaffold assembly

Cylindrical 3D scaffolds were prepared by mixing fibrinogen at 50 mg/ml (Sigma-Aldrich), α-aprotinin 15,600 U/ml (Sigma-Aldrich), and α-MEM (Corning, NY, United States) containing 1 × 10^6^ cells. Two cell suspension solutions were prepared, composed by *h*BM-MSCs and *h*SkMs with PBMCs ([*h*BM-MSCs + *h*SkMs]: PBMCs, 1:1) and without PBMCs. This ratio was chosen based on previously published literature showing that lower PBMCs:co-cultured cells ratios are preferable (Sturlan et al., 2009; Zhang et al., 2015).

Suspensions were pipetted in different wells of a 96-well plate, thrombin at 100 U/ml (Sigma-Aldrich) was added, and samples incubated at 37°C for 30 min to allow fibrinogen polymerization. 3D scaffolds were then transferred to a culture plate (Supplementary Figure S2C).

### 2.7 Dynamic culture system by perfusion bioreactor

3D scaffolds were placed in a perfusion bioreactor, formed by a custom multi-well plate, milled in poly (methyl methacrylate) (PMMA, Altuglas^®^ CN 100 10000, Altuglas International, La Garenne-Colombes Cedex, FR), a biocompatible material for biomedical application (Samavedi et al., 2014). This plate has two holes allowing the insertion of silicon tubes (Tygon^®^, FR) for medium flowing at a constant flow rate of 1.0 ml/min maintained by peristaltic pumps (Pasini et al., 2019). This bioreactor system operates within a standard cell culture incubator (Supplementary Figure S2D).

### 2.8 Live and dead assay

Cell viability was detected by fluorescence live and dead assay after preparation (day 0) and at each time point (day 7, 14, and 21). Calcein AM solution (Cat. No C1359, Sigma-Aldrich) was used to stain live cells, while cell membrane-impermeable Ethidium homodimer I solution (Cat. No E1903, Sigma-Aldrich) for nuclei of dead cells. Cells were incubated for 1 h at 37°C, then washed in 1X PBS, and imaged using a fluorescence microscope (Eclipse Ti Nikon Corporation, Tokyo, Japan). Signal intensity was quantified using ImageJ software. Original RGB images were converted to 8-bit (grey scale) format, and tagged area intensities were expressed as mean value of pixel intensity within a range from 0 (dark) to 255 (white), as previously reported (Spaepen et al., 2011).

### 2.9 RNA isolation and gene expression profiling

Expression of myogenic genes, including *Pax3*, *Myo D1*, My*f5*, *Myf6*, *Desmin*, and *MYH2* (Bio-Rad, Foster City, CA, United States), and cytokines, such as *IL6*, *TNF*, *IL12A*, *IL1B*, *IFNG*, *IL10*, *IL4*, *TGFB1*, and *TGFB2* (sequences are reported in Table 1) were investigated by reverse transcription quantitative polymerase chain reaction (RT-qPCR).

**TABLE 1:** Cytokine RNA sequences.

Gene symbol	Gene bank accession number	Sequences	Product size	Primer efficiency (%)
*IL6*	NM-000600.5	Forward: ACT​TGC​CTG​GTG​AAA​ATC​AT	135	106
		Reverse: CAG​GAA​CTG​GAT​CAG​GAC​TT		
*TNF*	NM-000594.4	Forward: GCC​CAT​GTT​GTA​GCA​AAC​CC	97	105
		Reverse: TAT​CTC​TCA​GCT​CCA​CGC​CA		
*IL12A*	NM-000882.4	Forward: TCA​GAA​TTC​GGG​CAG​TGA​CT	163	110
		Reverse: AGTCCCAQTCCTTCTTTCCCC		
*IL1B*	NM-000576.3	Forward: GGA​GAA​TGA​CCT​GAG​CAC​CT	185	110
		Reverse: GGA​GGT​GGA​GAG​CTT​TCA​GT		
*IFNG*	NM-000619.3	Forward: TGC​AGA​GCC​AAA​TTG​TCT​CC	194	110
		Reverse: TGC​TTT​GCG​TTG​GAC​ATT​CA		
*IL10*	NM-000572.3	Forward: AAG​ACC​CAG​ACA​TCA​AGG​CG	85	110
		Reverse: AAT​CGA​TGA​CAG​CGC​CGT​AG		
*IL4*	NM-000589.4	Forward:CTGCTTCCCCCTCTGTTCTTC	117	110
		Reverse:TTCGCTCTGTGAGGCTGTT		
*TGFB1*	NM-000660.7	Forward: GCA​CTC​GCC​AGA​GTG​GTT​AT	81	95
		Reverse: AAG​CCC​TCA​ATT​TCC​CCT​CC		
*TGFB2*	NM-0001135599.4	Forward: CCCTAAGCGCAATTCCAC	213	106
		Reverse: CTG​CTC​CTC​CTT​CTC​TTG​CT		

Total RNA from 3D dynamic cultures at each time point was extracted using QIAzol Lysis Reagent (Qiagen), chloroform (Sigma-Aldrich), and RNeasy Micro Kit (Qiagen). For each sample, 1 μg of total RNA was reverse transcribed using iScript™ cDNA synthesis kit (Bio-Rad), and relative gene expression analysis was performed on a LightCycler^®^ 480 Instrument (Roche, Basel, Switzerland) using SsoAdvanced™ universal SYBR^®^ Green Supermix (Bio-Rad). Specificity of formed products was assessed by melting curve analysis. Experiments were run in triplicate.

Data were normalized to glyceraldehyde-3-phosphate dehydrogenase (*GAPDH*) expression (reference gene) applying the geNorm method (Hellemans et al., 2007) and using CFX Manager software (M < 0.5). Fold changes were determined by 2^−ΔΔCt^ method and presented as relative levels *versus h*SkMs and *h*BM-MSCs at day 0. All experiments were performed in biological triplicates (N = 3), and each experiment in technical triplicate.

### 2.10 Immunofluorescence assay

Fibrin scaffolds were fixed in 4% PFA for 2 h at RT, cryo-protected in 30% sucrose (4 °C, overnight), included in optimal cutting temperature (OCT) compound, and cut in slices of 10 µm thickness using a cryostat (CM 1950, Leica, Wetzlar, Germany). Slices were permeabilized with 0.1% Triton X-100 for 10 min and blocked with horse serum solution for 1 h. Samples were then stained for Desmin (1:100; Abcam) and MYH2 (1:50, Thermo Fisher Sci.), incubated overnight at 4°C, and subsequently incubated for 1 h at RT with Alexa Fluor ™ 488 goat anti-rabbit IgG (1:400; Thermo Fisher Sci.), VectaFluor™ anti-mouse IgG Dylight 594^®^ kit (Vector laboratories), and DAPI. Slices were also stained for FITC-conjugated CD90 and PE-conjugated CD105 (Beckman Coulter), incubated overnight at 4°C, and the cell nuclei were counterstained using DAPI. Separate images were acquired using identical settings of light intensity, exposure time, and gain using a fluorescence microscope (Eclipse Ti Nikon Corporation, Tokyo, Japan).

### 2.11 Cytokine detection

For quantification of secreted cytokines in culture medium, an immunobead-based multiplex assay (Merck, Millipore) was employed for measurement of EGF, Eotaxin, GM-CSF, IFN-γ, IL-10, IL-12p70, IL-1RA, IL-1a, IL-1b, IL-2, IL-3, IL-4, IL-5, IL-7, MIP-1a, MIP-1b, TNF-α, bFGF, G-CSF, GRO, IL-6, IL-8, MCP-1, and VEGF, following manufacturer’s instructions.

### 2.12 Hematoxylin&Eosin staining

Scaffold slices (15 μm thickness) were hydrated using a decreasing ethanol gradient, washed for 5 min in water and incubated with hematoxylin and eosin for 60 min, then dehydrated using an increasing ethanol gradient and cleared in xylene for 5 min. Sections were mounted using Eukitt (Sigma-Aldrich) mounting medium. Images were acquired using an Olympus microscope BX53 equipped with ProgRes SpeedXT ^core^ five camera.

### 2.13 Statistical analysis

Data were analyzed using Prism software (v.9.0, GraphPad software, LLC, San Diego, California, United States). Results are presented as mean ± standard deviation (SD). Statistical analysis was performed using two-tailed independent Student’s t-test for two group comparisons, or two-way analysis of variance (ANOVA) test for three or more group comparison with Tukey’s test for multiple comparisons between group. For flow cytometry data, results are presented as percentage of positive cells, and expression of each marker on single cells is also reported as histograms and using unstained samples as negative controls. For characterization of mesenchymal cells in the co-culture system, percentage of positive cells and median fluorescence intensity (MFI) values were calculated for each marker. Surface marker expression variations were calculated as fold change normalizing MFI values for each marker and from each time point to MFI obtained from *h*BM-MSC cultured alone (Tsai et al., 2020). A *p*-value < 0.05 was considered statistically significant (de Winter 2013).

## 3 Results

### 3.1 3D scaffold optimization

Different concentrations of fibrinogen (50 and 100 mg/ml) were tested to check the best concentration to simultaneously assure good scaffold integrity and void spaces with totally interconnected cells within 3D fibrin system. Fibrinogen concentrations of 5 mg/ml was too low to induce polymerization, whereas, scaffold obtained at 20 mg/ml were difficult to be managed and were discharged (unpublished data). To better observe internal scaffold morphology obtained at 50 and 100 mg/ml, hydrogels were converted to aerogels by dense gas drying process, a procedure already documented as capable to avoid the natural shrinkage and collapsing of the 3D hydrated system, normally observed in lyophilization, as previously optimized (Della Porta et al., 2013). Indeed, derived alcohol gels were processed with dense carbon dioxide for 4 h at 38°C and 200 bar with a flow rate of 1.2 kg/h (system technology scheme in Supplementary Figure S2B). Collected aerogels maintained the same volume with a shrinkage <2%. Thanks to this extremely low shrinkage scaffold morphology was investigated after their freeze-fracture and the internal void structure was observed by FE-SEM (Figure 1). Scaffolds obtained using 50 mg/ml of fibrinogen showed thinner and wider meshes than those obtained with 100 mg/ml of fibrinogen. Pores of the 50 mg/ml of fibrinogen scaffold showed a mean perimeter of 548 nm and a mean Feret’s diameter of 180 nm, while at 100 mg/ml of fibrinogen, pores had smaller perimeter (mean, 128 nm) and Feret’s diameter (mean, 40 nm). Therefore, scaffolds made with 50 mg/ml of fibrinogen were chosen for further experiments, because they displayed a more spacious microenvironment suitable for a better cell distribution and viability.

### 3.2 Dynamic culture by perfused systems assures long-term viability

**FIGURE 2:**
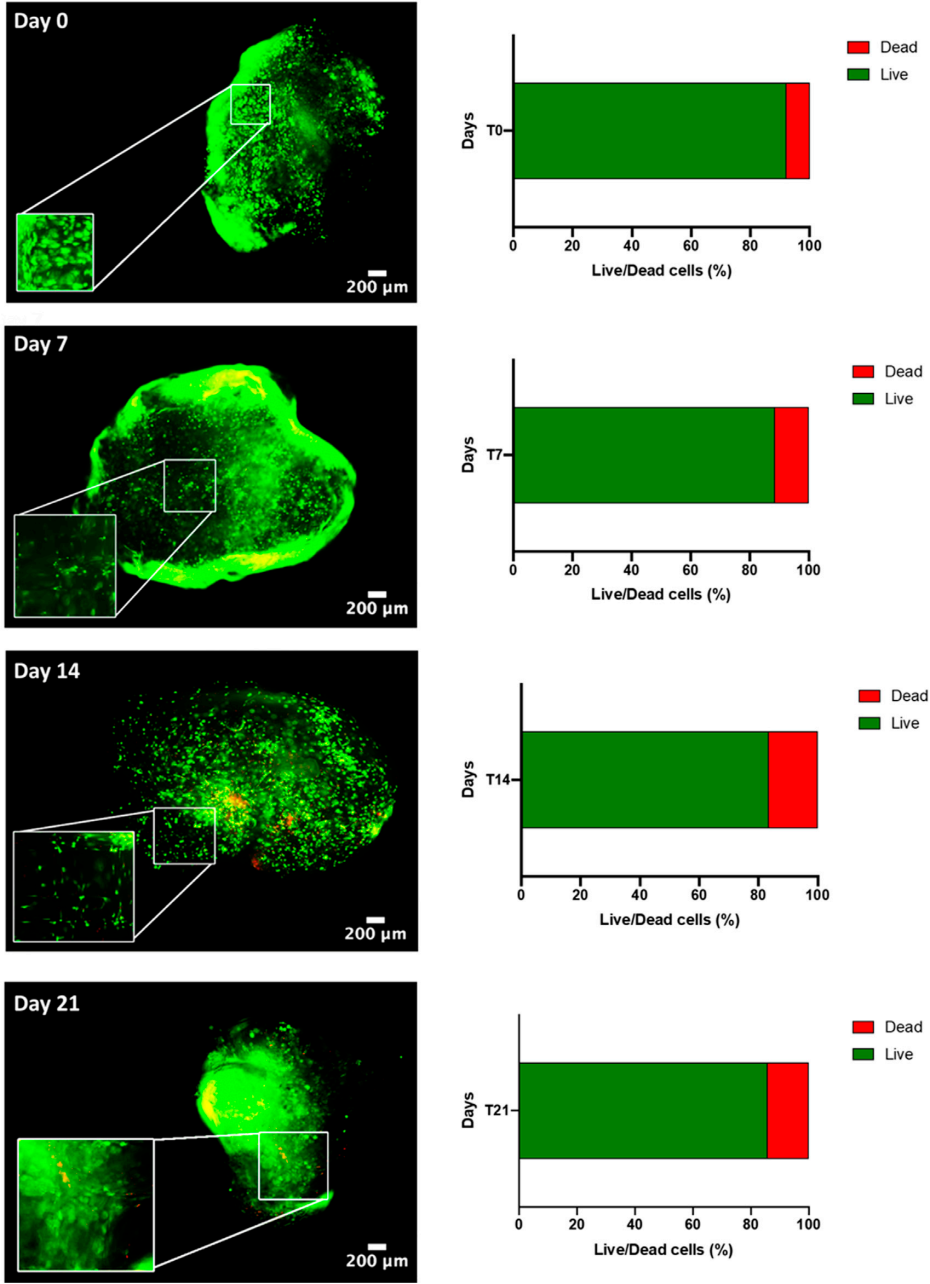
Live & Dead of *h*BM-MSC-*h*SkM-PBMC viability in a 3D fibrin scaffold cultured in perfusion system. Live cells are in green while dead cells in red. Images were captured at day 0, 7, 14 and 21 of culture, at ×4 magnification, scale bar: 200μm, while zoomed areas are at 200%. Histograms report viability quantification.

**FIGURE 3:**
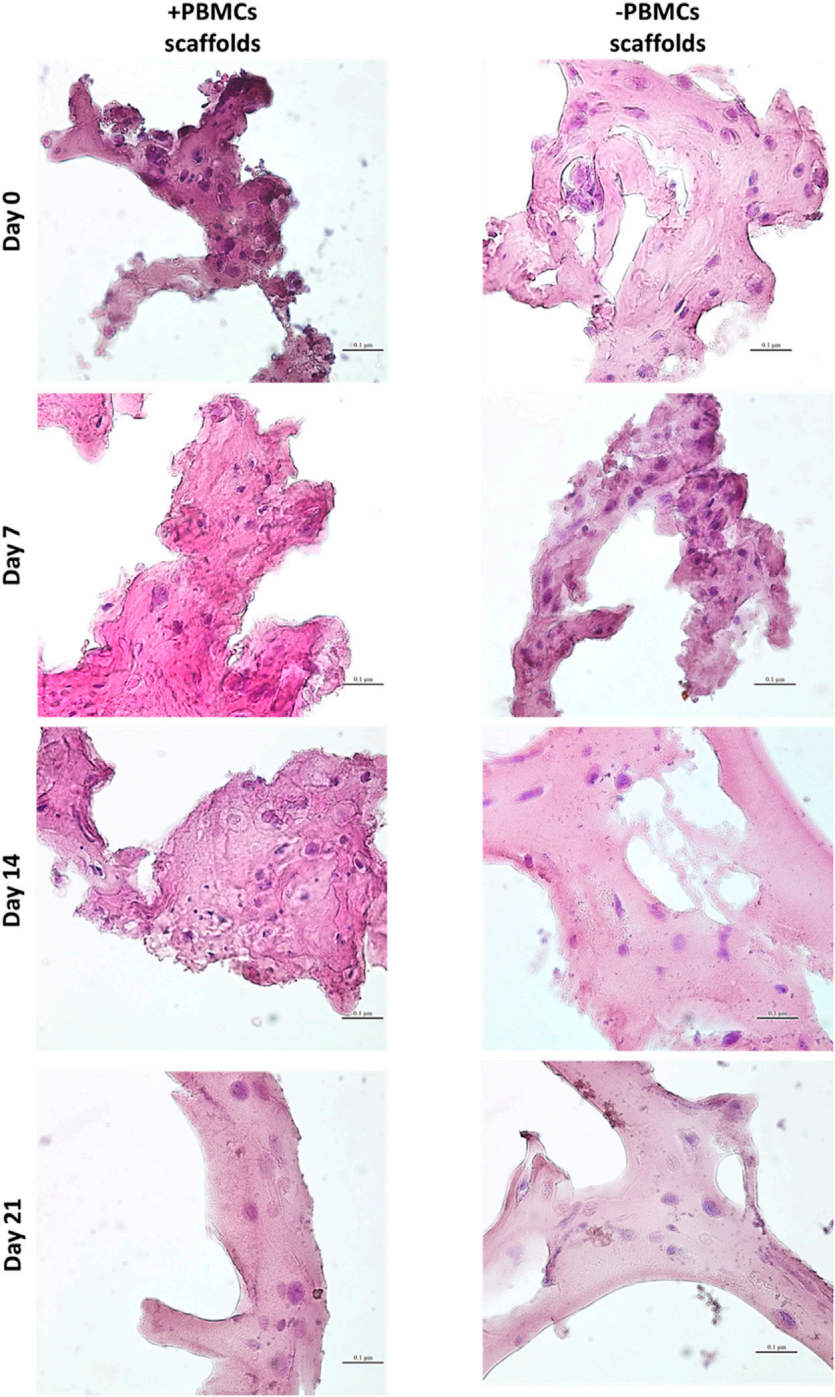
Hematoxylin & Eosin staining on 3D fibrin scaffolds embedded with *h*BM-MSCs-*h*SkMs with and without PBMCs. Images were acquired at day 7, 14 and 21 at ×40 magnification, scale bar: 20 µm.

Next, a myogenic commitment model of *h*BM-MSCs-*h*SkMs co-cultured (ratio 2:1) with or without PBMCs was established using scaffolds composed by fibrinogen at 50 mg/ml as support for cell culture and placed in dynamic conditions using a custom-made perfusion bioreactor for maintaining a constant medium flow rate of 1 ml/min (Della Porta et al., 2015) (Supplementary Figures S2C,D). To confirm suitability of this *in vitro* system before performing further experiments, cell viability within fibrin scaffolds was assessed by live and dead assay over a culture period of 21 days. As expected, total cells were almost represented by live cells (>80%) throughout the culture (Figure 2). Furthermore, fibrin scaffolds also maintained structure and integrity, as cells were homogenously distributed and were in close contact throughout the culture period. This regular distribution was also documented by histological evaluation of 3D scaffold culture slices using hematoxylin and eosin staining (Figure 3). Furthermore, fibrin matrix behaves as inert matrix not really influencing myogenic marker expression of *h*BM-MSCs-*h*SkMs co-culture; indeed, when, those cells were co-seeded in 2D culture, almost similar gene expression profile has been observed (Scala et al., 2022).

### 3.3 *h*BM-MSC and filtrated PBMC characterization

**FIGURE 4:**
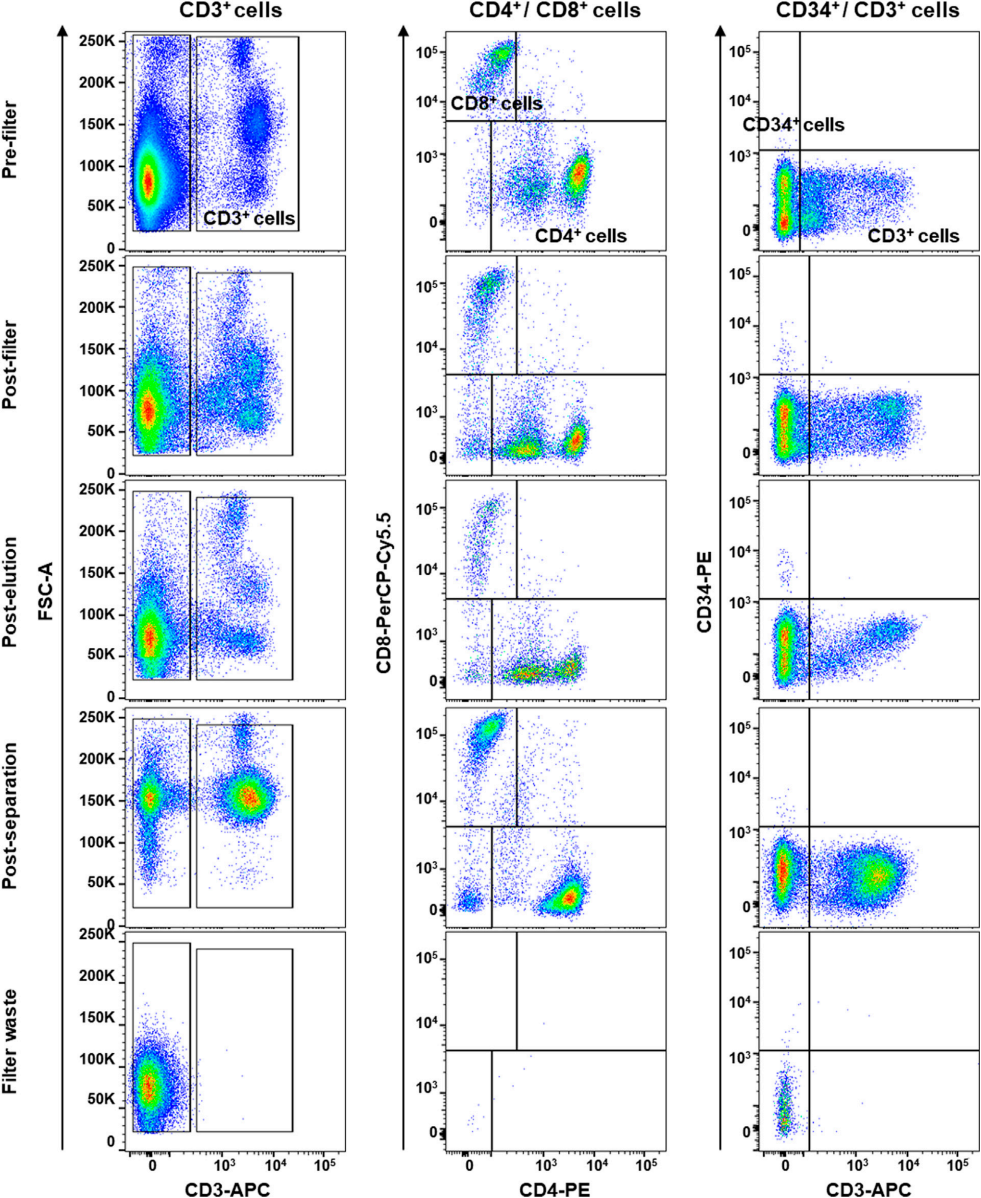
Flow cytometry immunophenotyping of peripheral blood mononuclear cells (PBMCs) obtained by whole blood was collected and filtered by a HemaTrate^®^ Blood Filtration system. PBMC different population fraction was subsequently separated by Ficoll-Paque density gradient separation to perform flow cytometry immunophenotyping on pre- and post-filtration, post-elution, post-separation, and on the filter bag waste, as negative control. Frequencies of CD3^+^ T lymphocytes and CD8^+^ and CD4^+^ subsets and circulating CD34^+^ hematopoietic stem cells were studied.

First, mesenchymal phenotype of our primary *h*BM-MSCs was confirmed following International Society of Cellular Therapy criteria (Dominici et al., 2006): adherence to tissue culture plastics; fibroblast-like shape; positivity for CD90, CD105, and CD73 mesenchymal markers; and negativity for CD34, CD14, CD45, and HLA-DR by flow cytometry (Supplementary Figure S1). Then, PBMCs were collected by filtration of 120 ml of whole blood adopting a system conventionally used in clinical practice (HemaTrate^®^ system), and nucleated cell fraction was concentrated in 10 ml of physiological solution. PBMC immunophenotyping and whole blood composition were investigated by flow cytometry on cells separated by Ficoll-Paque density gradient; filter waste bag was considered as a negative control for each donor employed (Figure 4). No significant variations were described in blood frequency and composition before and after filtration or separation, while no PBMCs were observed in specimens obtained from filter waste bags (Figure 4). Moreover, no differences were described for total CD3^+^ T Cells and CD4^+^/CD8^+^ subpopulations (all *p* > 0.05), and for circulating CD34^+^ hematopoietic stem cells (*p* = 0.2875), confirming that HemaTrate^®^ filter was highly efficient in selective PBMC collection and concentration in a 10 ml final volume of filtrate.

### 3.4 *h*BM-MSCs co-cultured with *h*SkMs and PBMCs in 3D dynamic conditions show a stable myogenic phenotype

**FIGURE 5:**
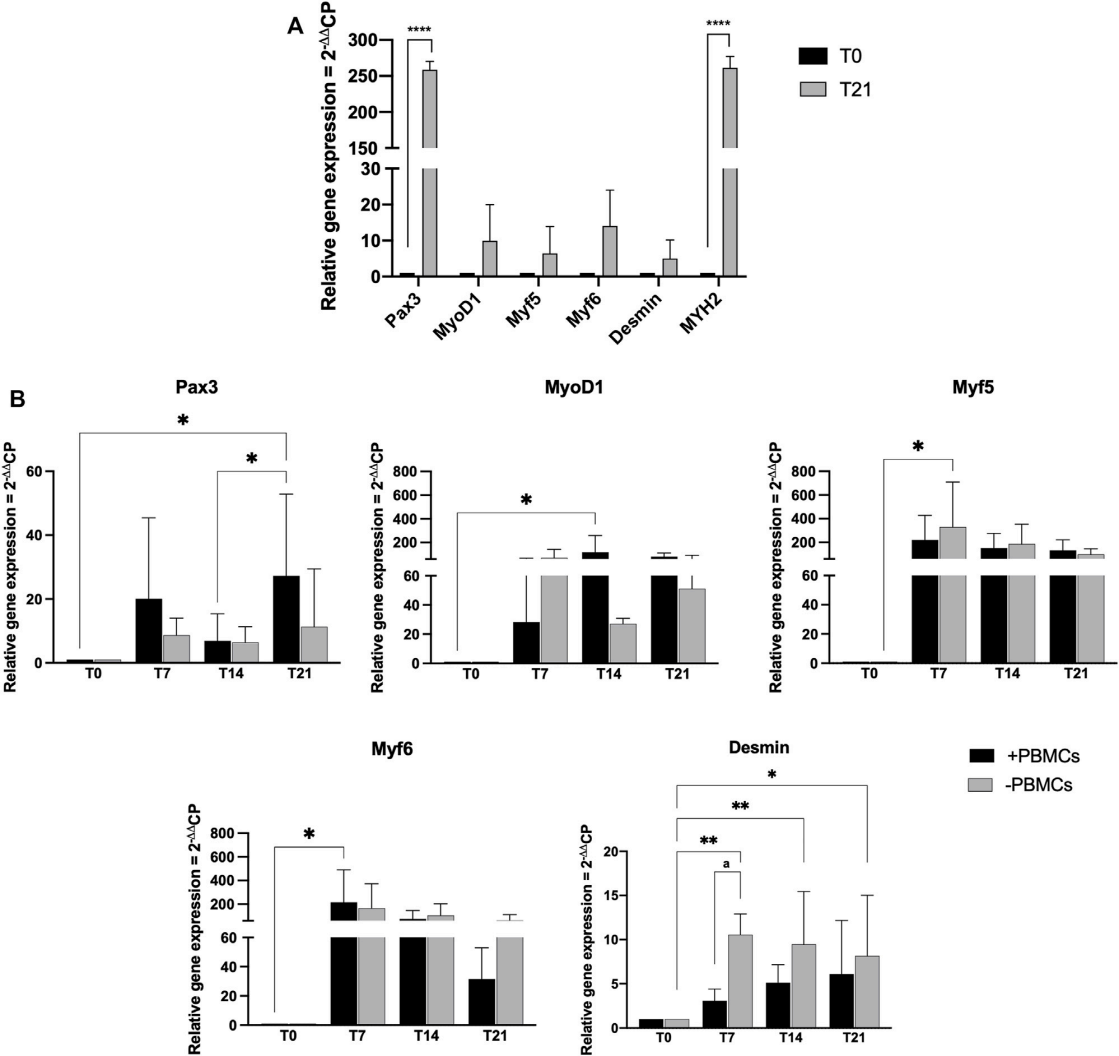
Gene expression profiling for myogenic markers by quantitative RT-PCR of *h*SkMs **(A)** and of *h*BM-MSCs-*h*SkMs-PBMCs **(B)**, both within 3D fibrin scaffold. mRNA levels of myogenic markers (*Pax3*, *MyoD1*, *Myf5*, *Myf6* and *Desmin*) were assayed by qRT-PCR at day 7, 14 and 21 of culture. Relative quantification of each mRNA gene expression normalized to endogenous *GAPDH* (internal control) was calculated using the 2^−ΔΔCt^ method and presented as fold change over *h*SkMs at day 0 **(A)** and over *h*BM-MSCs at day 0 **(B)**, selected as a control. All experiments were performed in biological triplicates (N = 3) and each experiment in technical triplicates. *, significant compared to day 0 (T0); a, significant comparison between ± PBMCs at day 7. *p < 0.05; **p < 0.01; ****p < 0.0001.

Myogenic commitment potential of our 3D system was investigated by gene expression profiling (Figure 5). First, *h*SkMs behavior in 3D culture was explored, as shown in Figure 5A *h*SkMs showed a significant upregulation of *Pax3* and *MYH2* at day 21 (258-fold and 261-fold, respectively, *p* < 0.0001), as well as *MyoD1*, *Myf5*, *Myf6*, and *Desmin* genes with an increasing trend from day 0–21. This behavior was reported for comparison purpose.

Then, in Figure 5B gene expression profiling of *h*BM-MSCs*-h*SkMs in 3D co-culture with and without PBMCs is showed*. Pax3* expression levels significantly differed between the two culture conditions over time. In details, *Pax3* expression was upregulated at day 7 in the presence of PBMCs (20-fold), while decreased at day 14 (7-fold), and significantly upregulated at day 21 (27-fold, *p* < 0.05). A similar trend was described in the absence of PBMCs (from 8.6-fold at day 7 to 6.4-fold at day 14 and 11.3-fold at day 21).

For MRF genes, *MyoD1* expression showed lower fold-change values than those without PBMCs at day 7 (28.35-fold vs*.* 70-fold); however, at day 14 in the presence of PBMCs a significant upregulation occurred (118-fold, *p* < 0.05) as well as at day 21 (81-fold vs*.* 51-fold). In cultures with PBMCs, *Myf5* expression was increased about 220-fold and without PBMCs about 329-fold (*p* < 0.05) at day 7, while at day 14 slightly decreased with PBMCs (152-fold with vs*.* 186-fold without PBMCs), remaining stable at day 21 (136-fold with vs*.* 99-fold without PBMCs). Generally, *Myf6* expression decreased along the culture with PBMCs, from 215-fold (*p* < 0.5) at day 7, to 75.2-fold at day 14, and 31-fold at day 21. Without PBMCs, the expression was of 165-fold at day 7, about 105-fold at day 14, and decreased at 64-fold at day 21. *Desmin* expression displayed an increasing trend with PBMCs about 3-fold at day 7, 5-fold at day 14, and 6-fold at day 21. When cultured without PBMCs, *Desmin* expression was slightly upregulated at day 7 and 14 (10-fold and 9.5-fold, respectively; *p* < 0.01), and at day 21 of 8-fold (*p* < 0.05). *MYH2* was also investigated but not detected at any time point along the culture. All these profiling were also different from those observed for the *h*SkMs alone in 3D culture, suggesting a fundamental role of the different cell population cross-talk within the 3D system.

**FIGURE 6:**
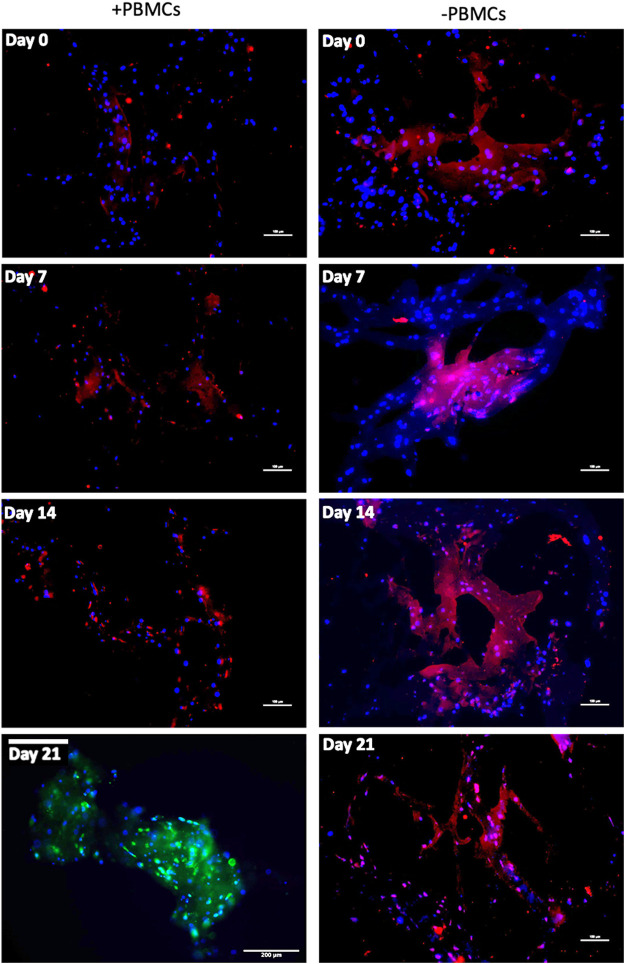
Immunofluorescence images of 3D fibrin scaffold for Desmin and MYH2 obtained by IF assay performed at day 7, 14 and 21 of culture. Desmin was stained in red and MYH2 in green; scaffolds embedded with *h*BM-MSCs-*h*SkMs-PBMCs (left column) and scaffolds embedded with *h*BM-MSCs-*h*SkMs (right column) are shown. MYH2 was not detected. All images were captured using ×20 magnification, scale bar: 200 µm.

**FIGURE 7:**
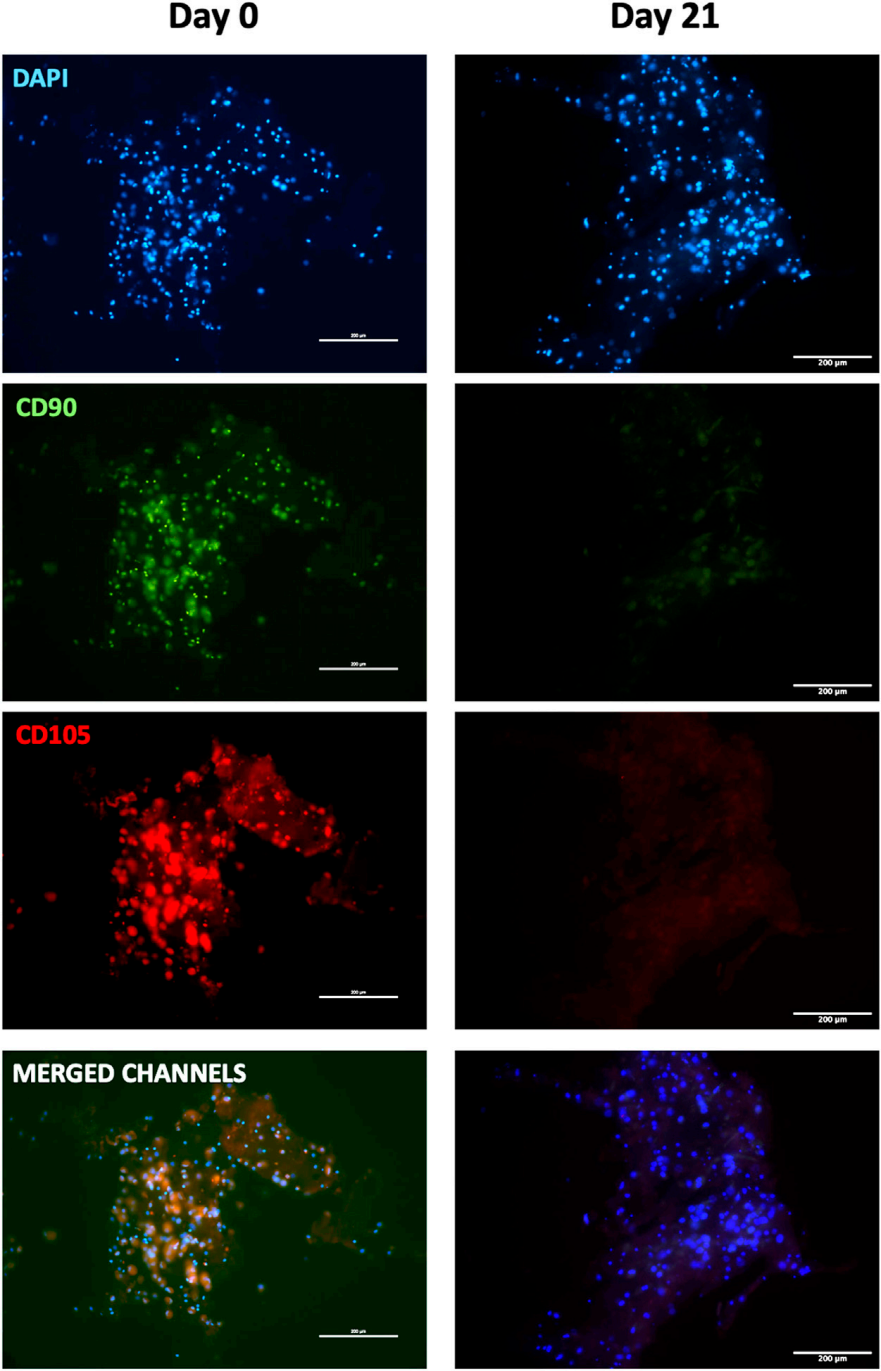
Immunofluorescence images of 3D fibrin scaffold for stemness markers by IF assay performed staining FITC-conjugated CD90 and PE-conjugated CD105 fibrin scaffolds embedded with *h*BM-MSCs-*h*SkMs-PBMCs at day 0 and 21. All images were captured using ×20 magnification, scale bar: 200 µm.

To further confirm myogenic commitment of *h*BM-MSCs with or without PBMCs in a 3D culture system, myogenic-related proteins, such as Desmin and MYH2, were assayed by IF (Figures 6, 7). In the presence of PBMCs, Desmin was detectable from day 7 with a signal that became brighter throughout the culture. Despite not well detected by qRT-PCR, MYH2 protein was highlighted by IF assay at day 21 days but only in the culture with PBMCs. Furthermore, almost all 3D cultures of *h*BM-MSCs-*h*SkMs without PBMCs displayed IF signals less intense at each time point compared to cultures with PBMCs (Figure 6). CD90 and CD105 protein expression was also investigated to confirm mesenchymal phenotype at baseline. Signals were clearly detected at day 0, confirming the mesenchymal phenotype of cultured *h*BM-MSCs, while very low or undetectable expression was observed at day 21 (Figure 7).

### 3.5 PBMCs influence cytokine expression

**FIGURE 8:**
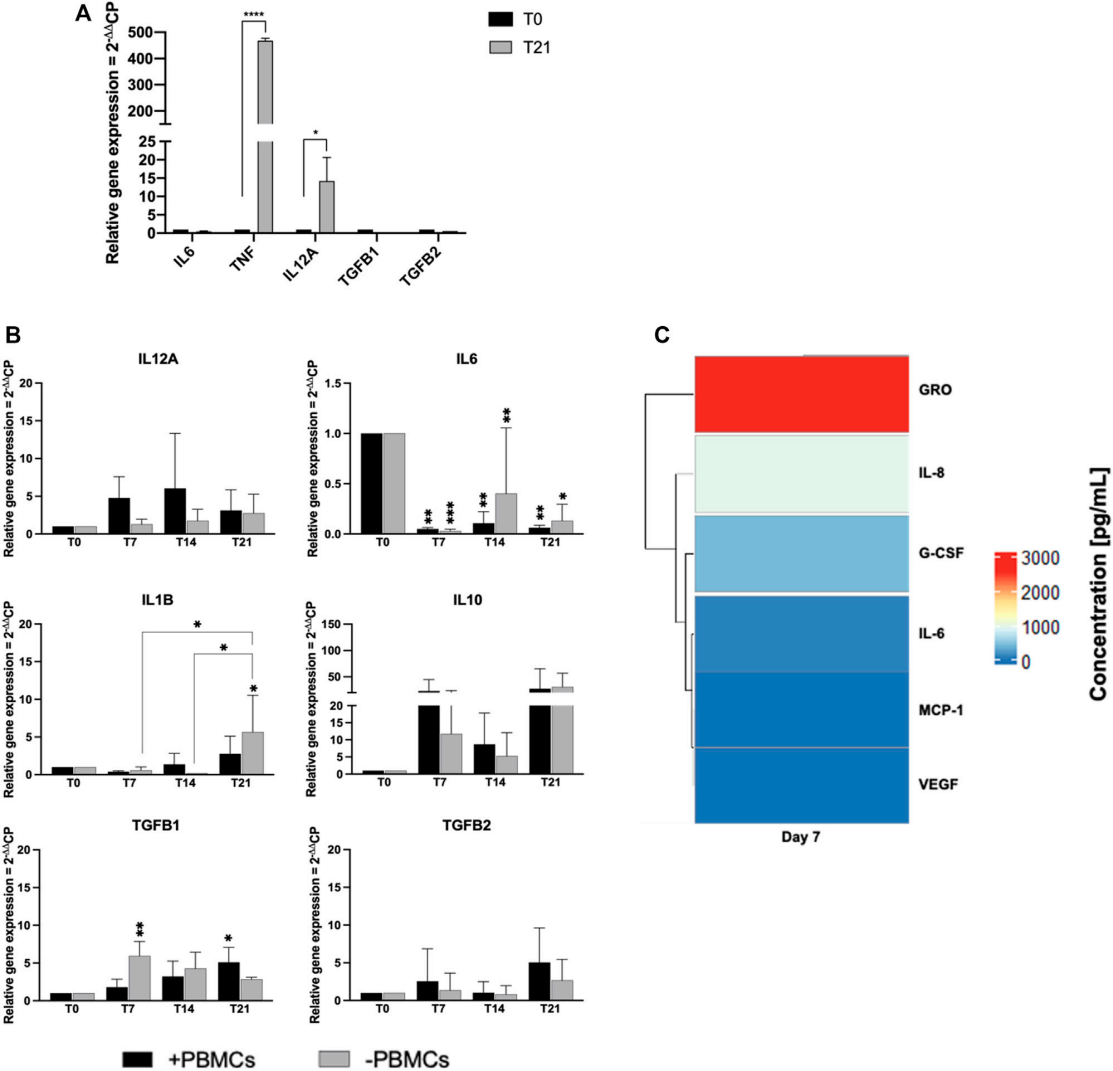
Gene expression profiling for pro- and anti-inflammatory cytokines by quantitative RT-PCR of *h*SkMs **(A)** and of *h*BM-MSCs-*h*SkMs-PBMCs **(B)**, both within 3D fibrin scaffold. The mRNA levels of pro-inflammatory cytokines (*IL12A*, *IL6* and *IL1B*) and anti-inflammatory cytokines (*IL10, TGFB1* and *TGFB2*) were assayed by qRT-PCR at day 7, 14 and 21 of culture. The relative quantification of each mRNA gene expression normalized to endogenous *GAPDH* (internal control) was calculated using the 2^−ΔΔCt^ method and presented as fold change over *h*SkMs at day 0 **(A)** and over *h*BM-MSCs at day 0 **(B)**, selected as a control. Cytokine levels measured in culture medium supernatants at day 7, 14, and 21 by magnetic bead-based multiplex immunoassay **(C)**. Data are reported as heatmap from blue (lowest value, 0 pg/ml of concentration) to red (highest value). Cytokines were hierarchical clustered based on expression pattern. Heatmap was made using *Pheatmap* and *ComplexHeatmap* packages in R Studio software (v. 2022.07.1 + 554; R Studio, Boston, MA, US). All experiments were performed in biological triplicates (N = 3) and each experiment in technical triplicates. *p < 0.05; **p < 0.01; ****p < 0.0001.

We next explored paracrine and cell-to-cell contact effects of PBMCs on cytokine expression in *h*BM-MSCs-*h*SkMs cultured in a 3D *in vitro* perfused model (Figure 8). *h*SkMs showed a significant upregulation of pro-inflammatory cytokine as *TNF*, 478-fold (*p* < 0.0001) and *IL12A,* 14-fold (*p* < 0.5) (Figure 8A). In co-culture system, pro-inflammatory cytokines were downregulated with PBMCs, especially *IL6* (0.04-fold at day 7, 0.1-fold at day 14, and 0.05-fold at day 21; *p* < 0.01). At day 14 and 21, *IL12A* increased of 6-fold and 17-fold with PBMCs, and of 75-fold and 13-fold without PBMCs; similarly, *IL1B* increased at 6-fold and 15-fold with PBMCs, while 20.5-fold and 8-fold without PBMCs*.* Conversely, among investigated anti-inflammatory cytokines, *IL10* was the most expressed and was higher in the presence of PBMCs (23.3-fold vs*.* 11.5-fold at day 7, or 9-fold vs*.* 5-fold at day 14, with vs*.* without PBMCs, respectively). *TGFB1* showed an increasing trend in the presence of PBMCs from 1.8-fold at day 7 to 9.5-fold at day 14 and 12-fold at day 21. *TGFB2* displayed the maximum expression at day 14, about 7-fold without PBMCs*. TNF, IL4*, and *IFNG* were not detected (Figure 8B).

Cytokine secretion was also investigated by analyzing culture medium supernatants at 7, 14 and 21 days by multiplex bead-based immunoassay (Figure 8C). Among 24 cytokines studied, growth-regulated alpha protein (GRO; 2.5 ng/ml), IL-6, granulocyte-colony stimulating factor (G-CSF), IL-8, monocyte chemoattractant protein-1 (MCP-1), and vascular epidermal growth factor (VEGF; 0.1 ng/ml) were detected in culture medium at day 7. Remaining cytokines were not present in culture medium supernatants at day 7, and no cytokines were detected at day 14 and 21.

## 4 Discussion

The development of accurate *in vitro* myogenic commitment models from *h*BM-MSCs is still challenging, and the exact role of PBMCs in influencing myogenic events is under debate, despite clinical reports indicated that the pharmacological targeting of inflammation and immune functions results in faster healing processes. Spontaneous healing skeletal muscle capacity in case of severe injuries appears insufficient, and conventional injury management, including RICE protocol (rest, ice, compression and elevation), drug therapies with non-steroidal anti-inflammatory drugs and intramuscular corticosteroids do not provide optimal restoration to preinjury status (Longo et al., 2012). Therefore, biological treatments, like cell therapy are of high clinical interest. In this sense, the development of biomimetic *in vitro* SkMR models are precious tools to better understand the complex mechanism of muscle healing process, as recently reported using an *in vitro* model of myogenic commitment by co-culture *h*BM-MSCs with *h*SkMs (Scala et al., 2022).

In this work, we implemented our previous *h*BM-MSC-*h*SkM co-culture system with PBMCs in a 3D fibrin scaffold to investigate their potential roles in muscle injury resolution through paracrine and cell-to-cell contact effects. Indeed, indirect evidence of PBMCs contribution in muscle tissue regeneration is in the clinical efficacy of autologous peripheral cell transplantation in critic limb ischemia treatments (De Angelis et al., 2015). In this case, PBMCs are collected from the patient by blood filtration using a commercial filter device (HemaTrate^®^), are concentrated in a smaller volume, and are then reinfused without any external cell manipulation. However, the exact mechanisms underlying clinical efficacy of autologous filtered peripheral blood cell transplantation are still under debate. Here, we sought to investigate the role of PBMCs in myogenesis using a 3D biomimetic *in vitro* model where PBMCs were added to a myogenic model composed by a co-culture of *h*BM-MSCs and *h*SkMs.

Scaffold porosity is another important parameter when setting up a 3D scaffold-based *in vitro* system, as cells require an appropriate void volume to locate and to permit oxygen and mass exchange (Lovecchio et al., 2014; Lamparelli et al., 2021; Lovecchio et al., 2020). Fibrinogen concentration can affect internal porosity with formation of irregular geometric areas. At the lowest fibrinogen concentrations tested (e.g., 5 mg/ml), polymerization reaction did not occur, while scaffold obtained at 20 mg/ml were difficult to be managed (unpublished data). Therefore, 50 mg/ml concentration was chosen to build fibrin scaffold because scaffold structure showed larger porosity with higher interconnectivity, whereas, higher fibrinogen concentrations decreased the scaffold pore size diameters, even if an increase in tensile strength is documented by the literature (Chiu et al., 2012). However, because tensile strength was not the focus of our study, fibrinogen concentration at 50 mg/ml was selected for our 3D system, allowing a better nutrient and gas exchange, confirmed by excellent cell viability up to 21 days of culture. Furthermore, to assure proper oxygen and metabolite mass transfer into the 3D high-density culture, scaffolds were placed within a close system in which culture medium re-circulated at a constant flow rate of 1 ml/min, maintained by a perfusion bioreactor. Indeed, even in standard 2D conditions, dynamic cultures assured a better myogenic commitment, as previously shown in our optimized co-culture system. Fibrin is widely used in tissue engineering and derives from thrombin-mediated fibrinogen monomer polymerization. Fibrin is naturally degraded by serine protease, and α-aprotinin, a serine protease inhibitor, is usually added to fibrinogen mix to prevent fibrin scaffold degradation in *vitro* and *in vivo* conditions, as previously demonstrated (Thomson et al., 2013; Mühleder et al., 2018).

*h*BM-MSCs cultured in 3D fibrin scaffold displayed upregulation of myogenic genes with conserved kinetics, as *MRFs*, such as *Myf5* and *Myf6*, were expressed earlier than *Desmin*, upregulated at later time points (Asfour et al., 2018). When PBMCs were embedded in the 3D fibrin scaffold together with *h*BM-MSCs and *h*SkMs, *h*BM-MSCs differentiated toward myogenic phenotype, although less efficiently when in the absence of PBMCs. However, in both conditions, *h*BM-MSCs lost their mesenchymal for a mature myocyte phenotype with CD90^−^CD73^+^CD105^+/−^ (Gago-Lopez et al., 2014) and showed Desmin and Myosin Heavy Chain II protein production. In our immunofluorescence analysis, cells appeared in a spherical shape and immunofluorescence signals were detected as single dots, because cells in a 3D system were in close contact with fibrin and embedded in the pores, as already reported (Sun et al., 2012; Ahmed et al., 2015).

The use of complex co-cultures could create a bias in gene expression normalization, as different cell populations were embedded together in the same scaffold and could not be separately recovered for gene expression analysis. For this reason, cultures with single populations, including *h*BM-MSCs and *h*SkMs alone, were used as controls and for gene expression normalization. Moreover, *h*SkMs were seeded at a lower density compared to *h*BM-MSCs (seeding ratio, 2:1, *h*BM-MSCs:*h*SkMs) in our 3D fibrin system, as previously optimized (Scala et al., 2022) thus, gene expression from co-culture experiments can be reconducted to *h*BM-MSC behavior. PBMCs were added to this well-established myogenic model at the lowest possible ratio (1:1), as lower PBMC ratios are the optimal solution in co-culture systems (Sturlan et al., 2009; Zhang et al., 2015).

Based on our results, PBMCs played an effective role in myogenic commitment, while their main activity was the influence on cytokine expression by *h*BM-MSCs. Fibrin can also induce synthesis of pro-inflammatory cytokines, especially from PBMCs (Jensen et al., 2007); however, pro-inflammatory cytokines were detected at very low levels in our 3D system throughout the culture period at both gene and protein levels, suggesting that fibrin scaffold did not influence gene and protein expression of BM-MSCs. Moreover, in our system, *TGFB2* was increased, contributing to a transient matrix deposit (Osses and Brandan, 2002; Ge et al., 2011), while *IL-10* upregulation*,* especially in the first 14 days of culture at gene and protein levels, could suggest a possible anti-inflammatory paracrine effect of PBMCs on microenvironment composition, promoting *h*BM-MSC commitment (Khodayari S., 2019). Secretion of chemoattracts and growth factors in culture medium could co-adiuvate physiological myogenic differentiation by triggering cell recruitment and neovascularization.

To our knowledge, this is the first study using a complex co-culture system composed by mesenchymal stem cells, muscle cells, and immune cells in a 3D microenvironment, representing a novelty in the tissue engineering field. In this *in vitro* myogenic model, immune cells exerted their effects only in a paracrine manner, while not influencing myogenic gene expression. Indeed, immune cells could reduce pro-inflammatory cytokines early in the healing process, while later induce anti-inflammatory molecules favoring muscle cell differentiation and regeneration (Tidball, 2017).

## 5 Conclusion and perspectives

The present work described the study of myogenic commitment of *h*BM-MSCs using a 3D scaffold of fibrin hydrogel bioengineered with different cell populations such as *h*SkMs and PBMCs in a perfused bioreactor system. The proposed system could be successfully employed for further studies on more complex crosstalk mechanisms involving multiple cell types. Furthermore, our *in vitro* biomimetic 3D model could allow to better understand the role of PBMCs in myogenic events in both physiological and pathological simulated conditions. Indeed, despite several clinical reports indicated that filtrated PBMCs fraction can be potentially useful in case of muscle severe injuries, biological mechanisms underlying stem cell differentiation and muscle regenerative events are still poorly understood. In this sense, the described 3D *in vitro* model might open perspectives for further exploration of the role of PBMCs behavior in myogenic healing events.

## Data Availability

The original contributions presented in the study are included in the article/Supplementary Material, further inquiries can be directed to the corresponding author.

## References

[B1] Ahmed T. A. E. Ringuette R. Wallace V. A. Griffith M. (2015). Autologous fibrin glue as an encapsulating scaffold for delivery of retinal progenitor cells. Front. Bioeng. Biotechnol. 2, 85. 10.3389/fbioe.2014.00085 25692127 PMC4315092

[B2] Asfour H. A. Allouh M. Z. Said R. S. (2018). Myogenic regulatory factors: The orchestrators of myogenesis after 30 years of discovery. Exp. Biol. Med. (Maywood) 243, 118–128. 10.1177/1535370217749494 29307280 PMC5788151

[B3] Beier J. P. Bitto F. F. Lange C. Klumpp D. Arkudas A. Bleiziffer O. (2011). Myogenic differentiation of mesenchymal stem cells co-cultured with primary myoblasts. Cell. Biol. Int. 35, 397–406. 10.1042/CBI20100417 20946104

[B4] Belizário J. E. Fontes-Oliveira C. C. Borges J. P. Kashiabara J. A. Vannier E. (2016). Skeletal muscle wasting and renewal: A pivotal role of myokine IL-6. SpringerPlus 5, 619. 10.1186/s40064-016-2197-2 27330885 PMC4870483

[B5] Birru B. Mekala N. K. Parcha S. R. (2018). Improved osteogenic differentiation of umbilical cord blood MSCs using custom made perfusion bioreactor. Biomed. J. 41, 290–297. 10.1016/j.bj.2018.07.002 30580792 PMC6306301

[B6] Charville G. W. Cheung T. H. Yoo B. Santos P. J. Lee G. K. Shrager J. B. (2015). *Ex vivo* expansion and *in vivo* self-renewal of human muscle stem cells. Stem Cell Rep. 5, 621–632. 10.1016/j.stemcr.2015.08.004 PMC4624935 26344908

[B7] Chaweewannakorn C. Tsuchiya M. Koide M. Hatakeyama H. Tanaka Y. Yoshida S. (2018). Roles of IL-1α/β in regeneration of cardiotoxin-injured muscle and satellite cell function. Am. J. Physiology-Regulatory, Integr. Comp. Physiology 315, R90–R103. 10.1152/ajpregu.00310.2017 29513560

[B8] Chen S.-E. Jin B. Li Y.-P. (2007). TNF-α regulates myogenesis and muscle regeneration by activating p38 MAPK. Am. J. Physiology-Cell Physiology 292, C1660–C1671. 10.1152/ajpcell.00486.2006 PMC3099536 17151142

[B9] Cheng M. Nguyen M.-H. Fantuzzi G. Koh T. J. (2008). Endogenous interferon-γ is required for efficient skeletal muscle regeneration. Am. J. Physiology-Cell Physiology 294, C1183–C1191. 10.1152/ajpcell.00568.2007 18353892

[B10] Chiu C. L. Hecht V. Duong H. Wu B. Tawil B. (2012). Permeability of three-dimensional fibrin constructs corresponds to fibrinogen and thrombin concentrations. BioResearch Open Access 1, 34–40. 10.1089/biores.2012.0211 23515363 PMC3559212

[B11] Ciardulli M. C. Lovecchio J. Scala P. Lamparelli E. P. Dale T. P. Giudice V. (2021). 3D biomimetic scaffold for growth factor controlled delivery: An *in-vitro* study of tenogenic events on wharton’s jelly mesenchymal stem cells. Pharmaceutics 13, 1448. 10.3390/pharmaceutics13091448 34575523 PMC8465418

[B12] Ciardulli M. C. Marino L. Lovecchio J. Giordano E. Forsyth N. R. Selleri C. (2020). Tendon and cytokine marker expression by human bone marrow mesenchymal stem cells in a hyaluronate/poly-lactic-Co-glycolic acid (PLGA)/Fibrin three-dimensional (3D) scaffold. Cells 9, 1268. 10.3390/cells9051268 32443833 PMC7291129

[B13] De Angelis B. Gentile P. Orlandi F. Bocchini I. Di Pasquali C. Agovino A. (2015). Limb rescue: A new autologous-peripheral blood mononuclear cells technology in critical limb ischemia and chronic ulcers. Tissue Eng. Part C. Methods 21, 423–435. 10.1089/ten.tec.2014.0245 25341088

[B14] de Winter J. C. F. (2013). Using the Student’s t-test with extremely small sample sizes. Pract. Assess. Res. Eval. 18. 10.7275/E4R6-DJ05

[B15] Della Porta G. Del Gaudio P. De Cicco F. Aquino R. P. Reverchon E. (2013). Supercritical drying of alginate beads for the development of aerogel biomaterials: Optimization of process parameters and exchange solvents. Ind. Eng. Chem. Res. 52, 12003–12009. 10.1021/ie401335c

[B45] Della Porta G. Nguyen B.-N. B. Campardelli R. Reverchon E. Fisher J. P. (2015). Synergistic effect of sustained release of growth factors and dynamic culture on osteoblastic differentiation of mesenchymal stem cells: Sustained Growth Factor Release for Osteoblastic Differentiation. J. Biomed. Mat. Res. 103, 2161–2171. 10.1002/jbm.a.35354 25346530

[B16] Deng B. Wehling-Henricks M. Villalta S. A. Wang Y. Tidball J. G. (2012). IL-10 triggers changes in macrophage phenotype that promote muscle growth and regeneration. J. I. 189, 3669–3680. 10.4049/jimmunol.1103180 PMC3448810 22933625

[B17] Dominici M. Le Blanc K. Mueller I. Slaper-Cortenbach I. Marini F. C. Krause D. S. (2006). Minimal criteria for defining multipotent mesenchymal stromal cells. The International Society for Cellular Therapy position statement. Cytotherapy 8, 315–317. 10.1080/14653240600855905 16923606

[B18] Engel N. Fechner C. Voges A. Ott R. Stenzel J. Siewert S. (2021). An optimized 3D-printed perfusion bioreactor for homogeneous cell seeding in bone substitute scaffolds for future chairside applications. Sci. Rep. 11, 22228. 10.1038/s41598-021-01516-8 34782672 PMC8593024

[B19] Ergene E. Sezlev Bilecen D. Kaya B. Yilgor Huri P. Hasirci V. (2020). 3D cellular alignment and biomimetic mechanical stimulation enhance human adipose-derived stem cell myogenesis. Biomed. Mat. 15, 055017. 10.1088/1748-605X/ab95e2 32442983

[B20] Gago-Lopez N. Awaji O. Zhang Y. Ko C. Nsair A. Liem D. (2014). THY-1 receptor expression differentiates cardiosphere-derived cells with divergent cardiogenic differentiation potential. Stem Cell Rep. 2, 576–591. 10.1016/j.stemcr.2014.03.003 PMC4050474 24936447

[B21] Gamblin A. L. Renaud A. Charrier C. Hulin P. Louarn G. Heymann D. (2014). Osteoblastic and osteoclastic differentiation of human mesenchymal stem cells and monocytes in a miniaturized three-dimensional culture with mineral granules. Acta Biomater. 10, 5139–5147. 10.1016/j.actbio.2014.08.033 25196309

[B22] Garcia S. M. Tamaki S. Xu X. Pomerantz J. H. (2017). “Human satellite cell isolation and xenotransplantation,” in *Skeletal muscle development* methods in molecular biology. Editor Ryall J. G. (New York, NY: Springer New York), 105–123. 10.1007/978-1-4939-7283-8_8 28842905

[B23] Ge X. McFarlane C. Vajjala A. Lokireddy S. Ng Z. H. Tan C. K. (2011). Smad3 signaling is required for satellite cell function and myogenic differentiation of myoblasts. Cell Res. 21, 1591–1604. 10.1038/cr.2011.72 21502976 PMC3364732

[B24] Gilbert-Honick J. Iyer S. R. Somers S. M. Lovering R. M. Wagner K. Mao H.-Q. (2018). Engineering functional and histological regeneration of vascularized skeletal muscle. Biomaterials 164, 70–79. 10.1016/j.biomaterials.2018.02.006 29499437

[B25] Giordano R. Canesi M. Isalberti M. Isaias I. Montemurro T. Viganò M. (2014). Autologous mesenchymal stem cell therapy for progressive supranuclear palsy: Translation into a phase I controlled, randomized clinical study. J. Transl. Med. 12, 14. 10.1186/1479-5876-12-14 24438512 PMC3912501

[B26] Govoni M. Berardi A. C. Muscari C. Campardelli R. Bonafè F. Guarnieri C. (2017). An engineered multiphase three-dimensional microenvironment to ensure the controlled delivery of cyclic strain and human growth differentiation factor 5 for the tenogenic commitment of human bone marrow mesenchymal stem cells. Tissue Eng. Part A 23, 811–822. 10.1089/ten.tea.2016.0407 28401805

[B27] Grayson W. L. Marolt D. Bhumiratana S. Fröhlich M. Guo X. E. Vunjak-Novakovic G. (2011). Optimizing the medium perfusion rate in bone tissue engineering bioreactors. Biotechnol. Bioeng. 108, 1159–1170. 10.1002/bit.23024 21449028 PMC3077473

[B28] Hellemans J. Mortier G. De Paepe A. Speleman F. Vandesompele J. (2007). qBase relative quantification framework and software for management and automated analysis of real-time quantitative PCR data. Genome Biol. 8, R19. 10.1186/gb-2007-8-2-r19 17291332 PMC1852402

[B29] Huang Y.-C. Dennis R. G. Larkin L. Baar K. (2005). Rapid formation of functional muscle *in vitro* using fibrin gels. J. Appl. Physiology 98, 706–713. 10.1152/japplphysiol.00273.2004 15475606

[B30] Jensen T. Kierulf P. Sandset P. Klingenberg O. Joø G. Godal H. (2007). Fibrinogen and fibrin induce synthesis of proinflammatory cytokines from isolated peripheral blood mononuclear cells. Thromb. Haemost. 97, 822–829. 10.1160/TH07-01-0039 17479194

[B31] khodayari S. Khodayari H. Amiri A. Z. Eslami M. Farhud D. Hescheler J. (2019). Inflammatory microenvironment of acute myocardial infarction prevents regeneration of heart with stem cells therapy. Cell Physiol. Biochem. 53, 887–909. 10.33594/000000180 31749350

[B32] Kuang S. Kuroda K. Le Grand F. Rudnicki M. A. (2007). Asymmetric self-renewal and commitment of satellite stem cells in muscle. Cell 129, 999–1010. 10.1016/j.cell.2007.03.044 17540178 PMC2718740

[B33] Lamparelli E. P. Lovecchio J. Ciardulli M. C. Giudice V. Dale T. P. Selleri C. (2021). Chondrogenic commitment of human bone marrow mesenchymal stem cells in a perfused collagen hydrogel functionalized with hTGF-β1-Releasing PLGA microcarrier. Pharmaceutics 13, 399. 10.3390/pharmaceutics13030399 33802877 PMC8002618

[B34] Londhe P. Davie J. K. (2011). Gamma interferon modulates myogenesis through the major histocompatibility complex class II transactivator, CIITA. Mol. Cell. Biol. 31, 2854–2866. 10.1128/MCB.05397-11 21576360 PMC3133399

[B35] Longo U. G. Loppini M. Berton A. Spiezia F. Maffulli N. Denaro V. (2012). Tissue engineered strategies for skeletal muscle injury. Stem Cells Int. 2012, 1–9. 10.1155/2012/175038 PMC3216349 25098362

[B36] Lovecchio J. Jonsdottir-Buch S. M. Einarsdottir G. K. Kjartan Gislason M. Örlygsson G. Sigurjonsson O. E. (2014). Assessment of perfusion bioreactors system using μCT technology and 3D modeling methods. Biomed. Technik/Biomedical Eng. 59, 302–305. 10.1515/bmt-2014-4130

[B37] Lovecchio J. Pannella M. Giardino L. Calzà L. Giordano E. (2020). A dynamic culture platform enhances the efficiency of the 3D HUVEC‐based tube formation assay. Biotechnol. Bioeng. 117, 789–797. 10.1002/bit.27227 31736057

[B63] Manzo P. Scala P. Giudice V. Gorrese M. Bertolini A. Morini D. (2022). c-Kit M541L variant is related to ineffective hemopoiesis predisposing to clonal evolution in 3D in vitro biomimetic co-culture model of bone marrow niche. Heliyon 8, e11998. 10.1016/j.heliyon.2022.e11998 36478848 PMC9720035

[B38] Matthias N. Hunt S. D. Wu J. Lo J. Smith Callahan L. A. Li Y. (2018). Volumetric muscle loss injury repair using *in situ* fibrin gel cast seeded with muscle-derived stem cells (MDSCs). Stem Cell Res. 27, 65–73. 10.1016/j.scr.2018.01.008 29331939 PMC5851454

[B39] Montarras D. Morgan J. Collins C. Relaix F. Zaffran S. Cumano A. (2005). Direct isolation of satellite cells for skeletal muscle regeneration. Science 309, 2064–2067. 10.1126/science.1114758 16141372

[B40] Mühleder S. Pill K. Schaupper M. Labuda K. Priglinger E. Hofbauer P. (2018). The role of fibrinolysis inhibition in engineered vascular networks derived from endothelial cells and adipose-derived stem cells. Stem Cell Res. Ther. 9, 35. 10.1186/s13287-017-0764-2 29433579 PMC5809876

[B41] Osses N. Brandan E. (2002). ECM is required for skeletal muscle differentiation independently of muscle regulatory factor expression. Am. J. Physiology-Cell Physiology 282, C383–C394. 10.1152/ajpcell.00322.2001 11788350

[B42] Pasini A. Lovecchio J. Ferretti G. Giordano E. (2019). Medium perfusion flow improves osteogenic commitment of human stromal cells. Stem Cells Int. 2019, 1–10. 10.1155/2019/1304194 PMC6525824 31191662

[B43] Persiani F. Paolini A. Camilli D. Mascellari L. Platone A. Magenta A. (2018). Peripheral blood mononuclear cells therapy for treatment of lower limb ischemia in diabetic patients: A single-center experience. Ann. Vasc. Surg. 53, 190–196. 10.1016/j.avsg.2018.05.036 30053546

[B44] Pollot B. E. Rathbone C. R. Wenke J. C. Guda T. (2018). Natural polymeric hydrogel evaluation for skeletal muscle tissue engineering. J. Biomed. Mat. Res. 106, 672–679. 10.1002/jbm.b.33859 28306190

[B46] Rigato M. Monami M. Fadini G. P. (2017). Autologous cell therapy for peripheral arterial disease: Systematic Review and meta-analysis of randomized, nonrandomized, and noncontrolled studies. Circ. Res. 120, 1326–1340. 10.1161/CIRCRESAHA.116.309045 28096194

[B47] Samavedi S. Poindexter L. K. Van Dyke M. Goldstein A. S. (2014). “Synthetic biomaterials for regenerative medicine applications,” in Regenerative medicine applications in organ transplantation (Elsevier), 81–99. 10.1016/B978-0-12-398523-1.00007-0

[B48] Scala P. Lovecchio J. Lamparelli E. P. Vitolo R. Giudice V. Giordano E. (2022). Myogenic commitment of human stem cells by myoblasts Co-culture: A static vs. a dynamic approach. Artif. Cells, Nanomedicine, Biotechnol. 50, 49–58. 10.1080/21691401.2022.2039684 35188030

[B49] Scala P. Rehak L. Giudice V. Ciaglia E. Puca A. A. Selleri C. (2021). Stem cell and macrophage roles in skeletal muscle regenerative medicine. IJMS 22, 10867. 10.3390/ijms221910867 34639203 PMC8509639

[B50] Shi Y. Wang Y. Li Q. Liu K. Hou J. Shao C. (2018). Immunoregulatory mechanisms of mesenchymal stem and stromal cells in inflammatory diseases. Nat. Rev. Nephrol. 14, 493–507. 10.1038/s41581-018-0023-5 29895977

[B51] Spaepen P. De Boodt S. Aerts J.-M. Sloten J. V. (2011). “Digital image processing of live/dead staining,” in *Mammalian cell viability* methods in molecular biology. Editor Stoddart M. J. (Totowa, NJ: Humana Press), 209–230. 10.1007/978-1-61779-108-6_21 21468981

[B52] Spaltro G. Straino S. Gambini E. Bassetti B. Persico L. Zoli S. (2015). Characterization of the Pall Celeris system as a point-of-care device for therapeutic angiogenesis. Cytotherapy 17, 1302–1313. 10.1016/j.jcyt.2015.04.006 26038175

[B53] Sturlan S. Sachet M. Baumann S. Kuznetsova I. Spittler A. Bergmann M. (2009). Influenza A virus induces an immediate cytotoxic activity in all major subsets of peripheral blood mononuclear cells. PLoS ONE 4, e4122. 10.1371/journal.pone.0004122 19125202 PMC2610492

[B54] Sun W. Tiemessen D. M. Sloff M. Lammers R. J. de Mulder E. L. W. Hilborn J. (2012). Improving the cell distribution in collagen-coated poly-caprolactone knittings. Tissue Eng. Part C. Methods 18, 731–739. 10.1089/ten.tec.2011.0593 22480276 PMC3460617

[B55] Tang H. Husch J. F. A. Zhang Y. Jansen J. A. Yang F. Beucken J. J. J. P. (2019). Coculture with monocytes/macrophages modulates osteogenic differentiation of adipose‐derived mesenchymal stromal cells on poly(lactic‐co‐glycolic) acid/polycaprolactone scaffolds. J. Tissue Eng. Regen. Med. 13, 785–798. 10.1002/term.2826 30771241 PMC6594112

[B56] Thomson K. S. Korte F. S. Giachelli C. M. Ratner B. D. Regnier M. Scatena M. (2013). Prevascularized microtemplated fibrin scaffolds for cardiac tissue engineering applications. Tissue Eng. Part A 19, 967–977. 10.1089/ten.tea.2012.0286 23317311 PMC3589898

[B57] Tidball J. G. (2017). Regulation of muscle growth and regeneration by the immune system. Nat. Rev. Immunol. 17, 165–178. 10.1038/nri.2016.150 28163303 PMC5452982

[B58] Tsai W. L. Vian L. Giudice V. Kieltyka J. Liu C. Fonseca V. (2020). High throughput pSTAT signaling profiling by fluorescent cell barcoding and computational analysis. J. Immunol. Methods 477, 112667. 10.1016/j.jim.2019.112667 31726053 PMC6981073

[B59] Vater C. Kasten P. Stiehler M. (2011). Culture media for the differentiation of mesenchymal stromal cells. Acta Biomater. 7, 463–477. 10.1016/j.actbio.2010.07.037 20688199

[B60] Villalta S. A. Rinaldi C. Deng B. Liu G. Fedor B. Tidball J. G. (2011). Interleukin-10 reduces the pathology of mdx muscular dystrophy by deactivating M1 macrophages and modulating macrophage phenotype. Hum. Mol. Genet. 20, 790–805. 10.1093/hmg/ddq523 21118895 PMC3024048

[B61] Witt R. Weigand A. Boos A. M. Cai A. Dippold D. Boccaccini A. R. (2017). Mesenchymal stem cells and myoblast differentiation under HGF and IGF-1 stimulation for 3D skeletal muscle tissue engineering. BMC Cell Biol. 18, 15. 10.1186/s12860-017-0131-2 28245809 PMC5331627

[B62] Zhang H. Shao B. Zhuge Q. Wang P. Zheng C. Huang W. (2015). Cross-talk between human neural stem/progenitor cells and peripheral blood mononuclear cells in an allogeneic Co-culture model. PLoS ONE 10, e0117432. 10.1371/journal.pone.0117432 25658950 PMC4319716

