# Supplementary material for: Peripheral blood mononuclear cells contribute to myogenesis in a 3D bioengineered system of bone marrow mesenchymal stem cells and myoblasts
Source: Front Bioeng Biotechnol. 2023 Jan 10;10:1075715. doi: 10.3389/fbioe.2022.1075715 (PMC9871311; doi:10.3389/fbioe.2022.1075715)
Supplement: Supplementary file 1 [file DataSheet1.pdf]

Supplementary Material

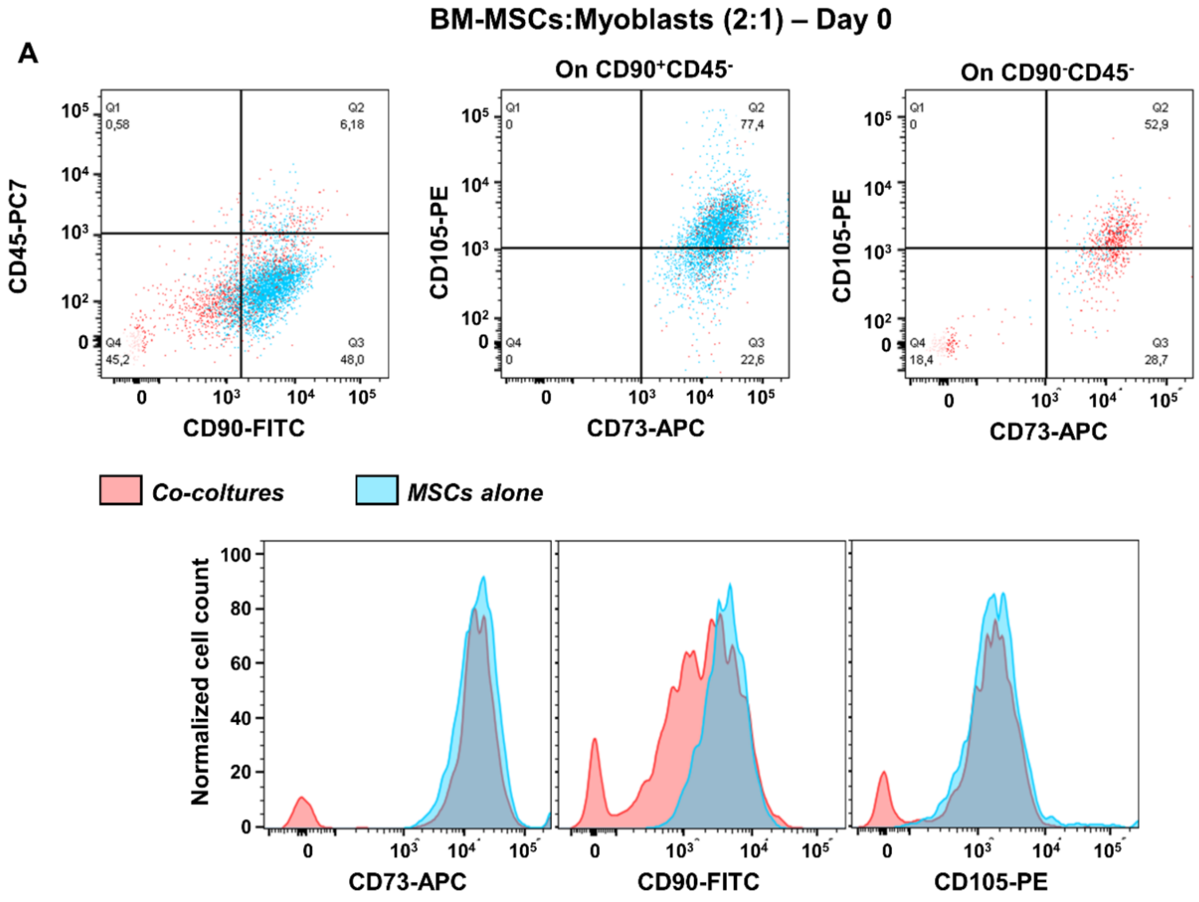

Supplementary Figure S1. Immunophenotype of *h*BM-MSCs-*h*SkMs co-culture at day 0 was performed by flow cytometry on *h*BM-MSCs-*h*SkMs co-culture and mesenchymal stem cells (MSCs) alone at day 0 (**A**), and CD90 expression was investigated on CD45$^-$ cells. Next, CD105 and CD73 expression was further studied on CD45$^-$CD90$^+$ and CD45$^-$CD90$^+$ cell populations (upper dot plots). Variations in marker expression are also displayed as normalized cell count histograms on single cells. On single cells, CD90 and CD45 expression was explored, and CD90$^+$CD45$^-$ cells were further studied for CD105 and CD73 expression. Similarly, on single cells, HLA-DR and CD34 expression was investigated, and CD34$^-$HLA-DR$^-$ cells were further studied for CD14 expression.

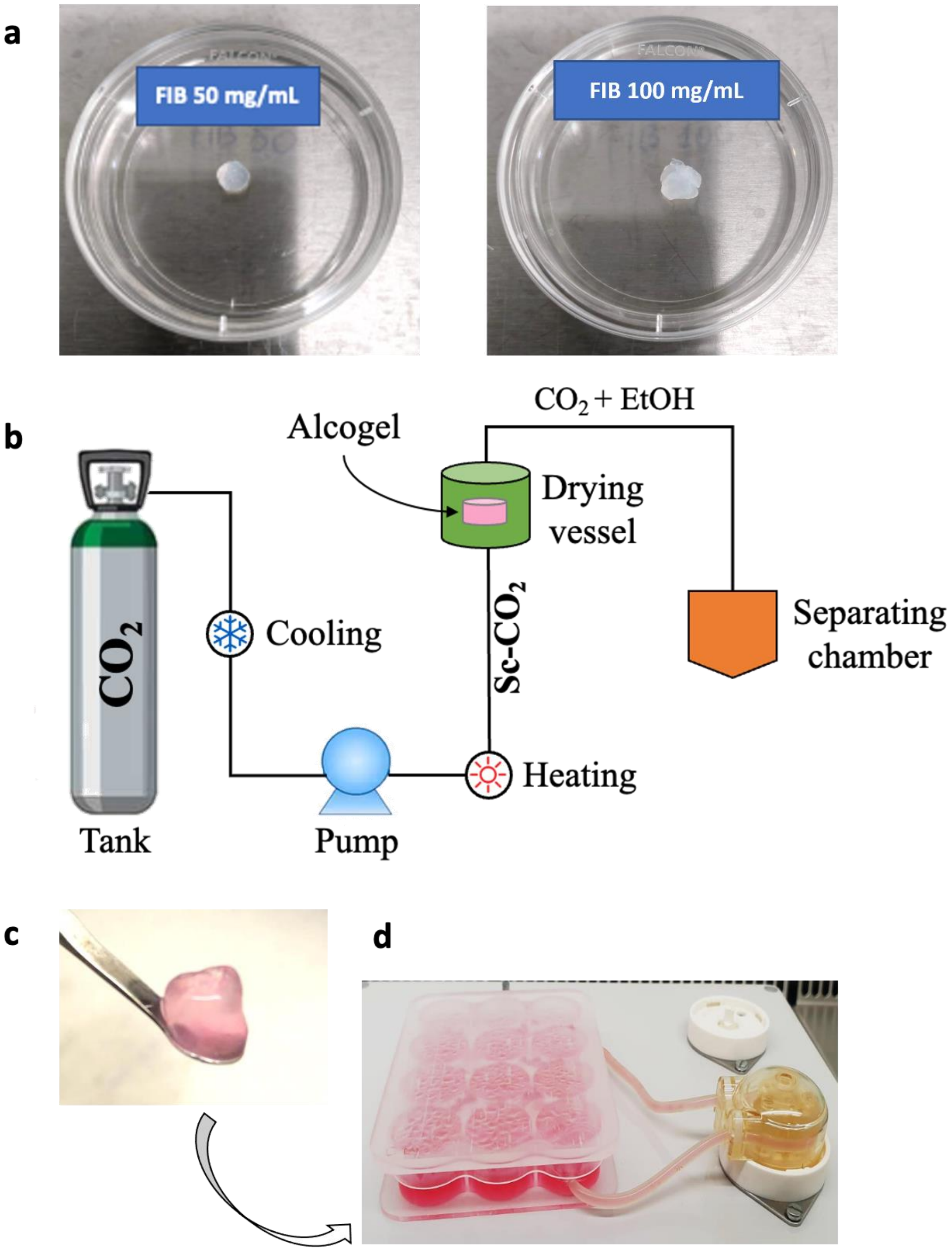

Supplementary Figure S2. Images of fibrin scaffold aerogels obtained at fibrinogen concentration of 50 mg/mL and 100 mg/mL after hydrogel processing by dense gas; aerogels were obtained, after conversion of hydrogel in alcohol-gel, then dried by dense carbon dioxide operating at 200 bar and 38°C **(a)**. Apparatus layout for scaffold drying using dense carbon dioxide: it consists of a high-pressure pump for dense carbon dioxide pumping and of a reactor in where the drying process is performed. Ethanol and carbon dioxide continuously flow through the reactor chamber and are collected in a separator located downstream **(b)**. Fibrin hydrogel scaffold image **(c);** perfusion bioreactor that allows a constant medium flow rate of 1 mL/min **(d)**.
